# Internet-Delivered Cognitive Behavioral Therapy for Problematic Alcohol Use in a Workplace Setting: Protocol for Quantitative and Qualitative Evaluation of Feasibility and Outcomes

**DOI:** 10.2196/18693

**Published:** 2020-07-15

**Authors:** David Forsström, Christopher Sundström, Anne H Berman, Kristina Sundqvist

**Affiliations:** 1 Department of Psychology Stockholm University Stockholm Sweden; 2 Centre for Psychiatry Research, Department of Clinical Neuroscience Karolinska Institutet & Stockholm Health Care Services Stockholm County Council Stockholm Sweden; 3 Department of Clinical Neuroscience Karolinska Institutet Stockholm Sweden; 4 Department of Psychology Uppsala University Stockholm Sweden

**Keywords:** workplace setting, ICBT, alcohol, protocol, mental health, feasibility, CBT, cognitive behavioral therapy, intervention, workplace

## Abstract

**Background:**

Internet-based cognitive behavioral therapy (ICBT) for mental health issues has been successfully implemented in routine health care settings, and research indicates that ICBT can also be applied to decrease problematic alcohol use in workplace settings. However, studies investigating the feasibility of implementing ICBT in a workplace setting have been lacking.

**Objective:**

The current study aims to investigate the feasibility of delivering ICBT for problematic alcohol use within an employee assistance program (EAP).

**Methods:**

The study has a quantitative naturalistic design, quantitively comparing ICBT and face-to-face treatment, and allowing for qualitative interviews with employees and employers. Recruitment of participants follows a five-session in-person psychological assessment at an EAP regarding an employee’s presumed problematic alcohol consumption. All assessed employees referred to ICBT or face-to-face treatment will be offered participation in the study. Interviews will be held with employees and their employer representatives following ICBT to elucidate both stakeholders’ experience and perception of ICBT and its context. Outcome comparisons between ICBT and face-to-face treatment will be assessed quantitatively using a Reliable Change Index and analysis of variance. Thematic analysis and Grounded Theory will be used to analyze the interview material.

**Results:**

The study is set to begin in April 2020 and to end in September 2021. The aim is to recruit up to 150 participants to the quantitative part of the study and 45 participants (15 employees and 30 employer representatives) to the qualitative part of the study.

**Conclusions:**

The current study will provide knowledge that is lacking and urgently needed on how to implement ICBT for problematic alcohol use in a workplace setting.

**International Registered Report Identifier (IRRID):**

PRR1-10.2196/18693

## Introduction

Problematic alcohol use causes harm both to the individual and to significant others, as well as generates considerable societal costs [1]. Alcohol consumption has declined in high-income countries, in parallel with substantial increases in lower-middle-income countries, although the overall prevalence of problematic alcohol use is still worryingly high [2]. In Sweden, 20% of men and 13% of women are considered hazardous drinkers [3]. Costs to society due to alcohol have been estimated to exceed one percent of the gross national product in high- and middle-income countries [1]. About half of all societal alcohol-related costs comprise loss of production at the workplace due to factors such as absence and reduced work capacity. Other common consequences of problematic alcohol use among employees are accidents, injuries, and increased healthcare costs [4-6]. The many negative consequences of hazardous alcohol use among employees are often presented as arguments for employers to implement alcohol prevention measures, capitalizing on the potential of the workplace to serve as a platform for primary, secondary, and tertiary prevention [7], since only one-fifth of individuals with alcohol use disorder seek treatment for their problems [8]. In Sweden, employment covers over two-thirds of the population between the ages of 15 and 74 [9]. Given that adults spend a large proportion of the day at the workplace, exposure to preventive interventions can be maximized [6]. To this end, employee assistance programs (EAP) have been developed to provide early and easily accessible help for employees [7].

Internet interventions in the form of internet-delivered cognitive behavior therapy (ICBT) have been studied for more than 20 years, and research shows that, for mood and anxiety disorders, they are often as effective as face-to-face psychotherapy [10]. Although some of these interventions have been developed as public health interventions, accessible to the general public, many have been developed as clinical alternatives to regular psychotherapy, and there are now several examples of clinics offering therapist-guided ICBT as an integrated part of routine health care [11]. Although there are fewer studies on ICBT for problematic alcohol use compared to ICBT for mood and anxiety disorders, the literature suggests that ICBT is also beneficial for this population [12]. A recent individual patient data meta-analysis demonstrated that internet interventions for problematic alcohol use are effective in reducing alcohol consumption, with guided interventions being more effective than unguided ones [13]. Studies on internet interventions for problematic alcohol use have mainly been conducted in general population samples [14], although there are successful examples of internet interventions conducted in clinical settings [15]. Further, a handful of studies have been conducted in workplace settings; for example, a six-week internet intervention specifically tailored to employees resulted in significant reductions in weekly standard units regardless of whether the employee received guidance from a psychologist or not [16], while a fully self-guided internet intervention with 62 modules, showed significant reductions over time in alcohol consumption. However, there were no significant differences in comparison to a self-help booklet [17]. Although these studies suggest that ICBT can be successfully used in a workplace setting, there are, to our knowledge, no studies on the feasibility of ICBT for employees at a site that provides EAP. Offering ICBT at sites using EAP has several advantages compared to face-to-face CBT. Since ICBT is available online anytime, the employee does not need to leave the workplace to receive treatment, thus reducing travel time and expenses associated with face-to-face treatment. If production loss due to treatment is cut, the employer might be more prone to pay for treatment and help the employee, particularly in smaller businesses and those not affiliated with an EAP provider [18]. Studies on the feasibility of ICBT at sites providing EAP, from both quantitative and qualitative perspectives, are needed in order to promote a greater understanding of how internet interventions might be provided in this context, possibly increasing the likelihood of successful dissemination of internet interventions in workplace settings in Sweden and elsewhere. Also, introducing ICBT at an EAP has several advantages compared to face-to-face treatment: lesser absenteeism from work due to reduced travel, less attrition from the more easily accessed treatment, and the employer might be more prone to cover treatment costs since cutting travel time means less production loss.

### Objectives

This study aims to investigate the feasibility of providing ICBT for employees with problematic alcohol use at Alna Sweden, a provider of EAP in Sweden. In order to determine feasibility, both quantitative and qualitative approaches will be used. The quantitative aspect of the study concerns evaluating ICBT in comparison with face-to-face treatment. The following research questions will be addressed:

1. What changes in alcohol consumption and mental health occur over time among employees who receive ICBT or face-to-face treatment for problematic alcohol use?
2. In what way do alcohol and mental health outcomes differ between employees exposed to ICBT as compared to face-to-face treatment?

The following research questions will be investigated in qualitative interviews among those receiving ICBT:

1. The employee perspective:
  - What does the employee perceive as positive or negative about receiving ICBT for problematic alcohol use?
  - Does the employee experience any specific influence of the workplace setting on receiving ICBT; if so, how is it expressed?
2. The employer perspective:
  - How does the employer perceive possible effects of ICBT on employee functioning in the workplace during and after treatment?
  - To what extent does the employer perceive ICBT as a feasible intervention for problematic alcohol use in the workplace setting?

## Methods

### Overview

**Figure 1:**
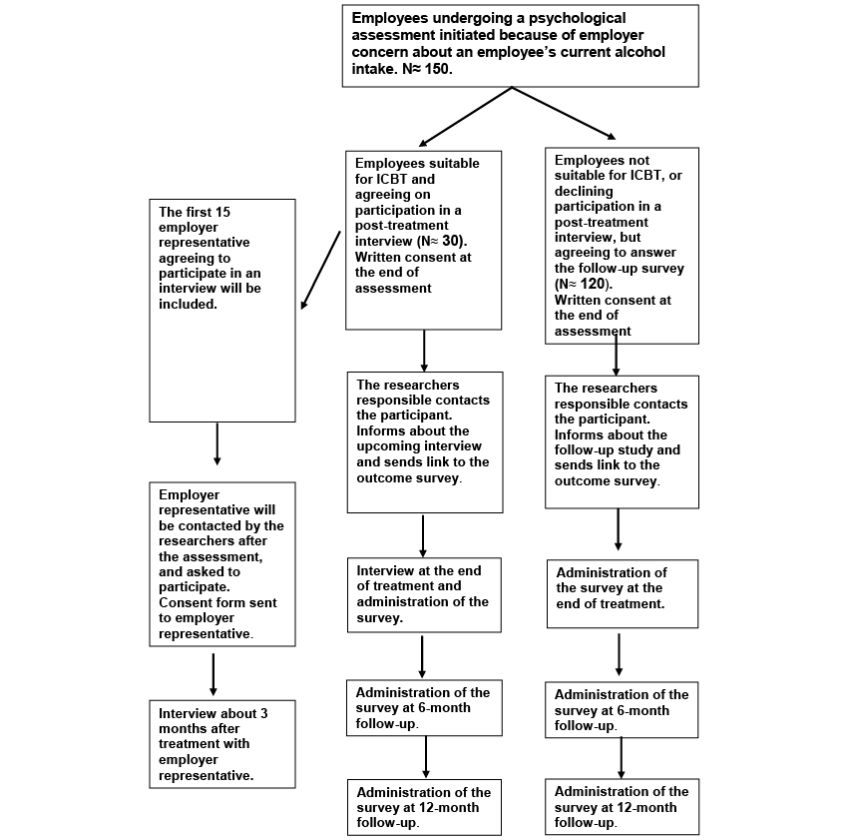
Flowchart for recruitment procedure.

In the current research project, employees who undergo an assessment at Alna Sweden and who are referred to psychotherapy for problematic alcohol use will be offered participation in the quantitative naturalistic study. Those who are recommended ICBT will also be offered participation in a qualitative study (see Figure 1 for an overview).

During the study, Alna will offer ICBT administered by treatment providers (psychologists and psychotherapists) working for Alna. The providers will receive training in ICBT delivery before the start of the study.

### Treatment Setting

Alna Sweden is an organization that was founded in 1961 by the central trade unions and employers’ organizations. The Alna EAP offers prevention, assessment, and treatment of alcohol and drug use problems among employees at organizations and companies all over Sweden. Alna conducts around 150 employee assessments per year. Usually, these assessments are initiated based on employer concern about an employee’s current alcohol or drug use. The Alna assessment, which is carried out by licensed psychologists, consists of a comprehensive mapping of the employee’s current problems and resources as well as an assessment of the employee’s treatment needs. In the majority of cases, the assessment leads to recommendations for further treatment, usually in the form of 10-15 sessions of face-to-face CBT based on relapse prevention [19]. In the vast majority of cases, the employer chooses to follow the recommendation for referral to treatment and also finances the treatment. With Sweden being a sparsely populated country, employees who receive treatment often live at great geographical distances from the nearest Alna Sweden treatment provider, resulting in significant workplace absenteeism in the form of travel time, aside from any absenteeism due to the actual session time.

### Recruitment Procedure

Recruitment will take place following the Alna assessment of employee needs. The assessment begins with a session where the employee participates together with an employer representative (usually the manager or personnel from human resources). The focus of this initial session is on the employee’s alcohol consumption, which has, in some way, affected work performance. The employee then undergoes a comprehensive psychological assessment over 3-4 subsequent sessions, which, in addition to alcohol consumption and other addictive behaviors, also covers mental and physical health, social relations, economic aspects, and motivation for change. Blood samples are taken in parallel to the psychological assessment.

### Naturalistic Study

Towards the end of the assessment, all employees referred for psychological treatment (both face-to-face and ICBT) will be invited to participate in the study by the Alna assessment psychologist. If the employee consents, the appropriate treatment (face-to-face or ICBT) will be proposed for the employer. If the employer approves the cost, the employee will be referred to the chosen treatment, and written informed consent will be obtained at the last assessment. The participants will then be contacted by the researchers and be asked to respond to an online survey, accessed via an e-mail link.

### Qualitative Study

In cases where the treatment provider considers ICBT a suitable for the employee, it will be presented as a treatment alternative requiring participation in post-treatment interviews. After that, the procedure is the same as in the naturalistic study. Hence, if the employer agrees to cover the cost for ICBT, and informed consent is collected, the participants will be contacted by the researchers and be asked to respond to the online survey, accessed via an e-mail link. The participants in the qualitative study will also receive information about the interview. These participants will be included in the naturalistic (responding to the survey) and the qualitative study (participating in the interviews). Up to 30 participants will be recruited for the qualitative study.

### Eligibility

#### Inclusion Criteria

All employees who have undergone the Alna assessment and have been referred to face-to-face treatment or ICBT are eligible to participate in the naturalistic study. Those who have been referred to ICBT are also eligible for inclusion in the qualitative study. ICBT will only be available to employees agreeing to participate in the qualitative study. All employers with employees who have been referred to ICBT will be offered participation in the part of the qualitative study that concerns employers.

#### Exclusion Criteria

For the naturalistic study, exclusion criteria are drug use (information collected during the assessment) and suicidality (information collected during assessment and treatment).

### Study Design

#### Naturalistic Study

All employees undergoing an Alna assessment and who are referred to psychological treatment, either regular face-to-face or ICBT, will be offered participation in the naturalistic study. Informed written consent will be provided at the final assessment session. Those who agree to participate will be provided a link to an online survey for pre-treatment measurement. A follow-up evaluation will take place immediately after completion of treatment (face-to-face psychotherapy or ICBT) and after another six and 12 months. Data collected at the start of the Alna assessment will be included retroactively, to allow evaluation of possible behavior changes during the assessment. Every employee that is offered treatment at Alna will be asked to participate in the study. Since Alna carries out approximately 150 assessments per year, the goal is to recruit all of the assessed employees. The recruitment process will go on for a year and a half if needed.

#### Qualitative Interview Study

In the qualitative study, employees referred to ICBT and their employers will be asked to participate in interviews, the former targeting the employee’s experiences of ICBT and the later targeting employer perceptions. A total of 45 participants will be recruited, with up to 30 employees and up to 15 employer representatives. Semi-structured interviews will take place within two months following treatment conclusion. Interview guides will be based on the research objectives presented. A panel of researchers with a background in addiction and/or internet-delivered treatment will be asked to review the interview guides to ensure that the relevant areas are covered. The employees and employer representatives who agree to participate in the study will receive the interview questions in advance to be able to reflect on the topics included, thus, increasing the possibility of describing their experiences of treatment accurately. The interviews will mainly be conducted via telephone or video meetings since participants are recruited from all over Sweden, but may also be carried out face-to-face. All of the interviews will be recorded and transcribed verbatim.

### Platform and Login Procedure for the Internet-Delivered Treatment

The web-based treatment platform that will be used to deliver ICBT will require a secure, two-factor identification bank-issued national e-identification login. This identification process ensures that the right person receives the treatment and that unauthorized persons cannot access the treatment or stored data.

### Outcome Measures

#### Online Survey Questionnaires

##### Timeline Followback

A seven-day Timeline Followback questionnaire (TLFB) [20,21] will be used to measure preceding week alcohol consumption, as measured in standard units. TLFB can be used during different measures of time, eg, one or two weeks. The user has to specify how much alcohol he/she has consumed each day during the specified period.

##### Alcohol Use Disorders Identification Test

The Alcohol Use Disorders Identification Test (AUDIT) will be used to measure problematic alcohol use [22]. AUDIT has 10 items. The minimum score is 0, and the maximum score is 40. Eight of the questions have an item response range from 0 to 4, ranging from “Never” to “Almost every day.” Two questions have an item response in three steps (0, 2, and 4), ranging from “Never” to “Almost every day.” The instrument has a one-factor solution.

##### Patient Health Questionnaire-9

The Patient Health Questionnaire-9 is a nine-item instrument that measures depression, including a final item measuring suicidal thoughts. The instrument has excellent internal consistency (Cronbach α=.89) and good test-retest correlation (.84) [23]. The instrument has a single-factor solution. The minimum score is 0, and the maximum is 27 points [23]. Item responses range from 0 (“Not at all”) to 3 (“Nearly every day”).

##### Generalized Anxiety Disorder-7

The Generalized Anxiety Disorder-7 is a short instrument designed to measure anxiety. Its internal consistency is excellent (Cronbach α=.92), and it has good test-retest reliability (intraclass correlation .83) [24]. The questionnaire contains seven questions and has a single-factor solution. It is a self-report measure, with a minimum score of 0 and a maximum score of 21 points, with an item range of 0 (“Not at all”) to 3 (“Nearly every day”) [24].

##### The World Health Organization-Five Well-Being Index

The World Health Organization-Five Well-Being Index is an internationally validated instrument focused on well-being, consisting of five items. Good internal consistency was observed in a Swedish general population sample (Cronbach α=.83). The minimum score is 0, and the maximum score is 15 [25]. Item responses range from 0 (“All of the time”) to 3 (“Never”).

### Blood Samples

Blood samples for two alcohol markers, carbohydrate-deficient transferrin (CDT) and Phosphatidylethanol (B-PEth), will be taken at the start of the assessment and then once a month during treatment. B-PEth and CDT are indicators of alcohol intake. Both markers can detect low levels of alcohol intake. The diagnostic sensitivity was 99% for B-PEth, and for CDT, the sensitivity varied based on intake from 40% for low intake to 90% for very high intake. Specificity is very high for PEth (99%) and is lower for CDT [26].

### Internet-Delivered Treatment

The ICBT program to be used in this study has been evaluated in previous studies in problematic alcohol use [27,28]. It is based on CBT and relapse prevention and includes 13 modules, each consisting of text and a worksheet with practical exercises. The program also includes an alcohol diary. In the current study, the program will be used together with guidance from a psychologist/psychotherapist both through written platform messages and via three video sessions. For details about treatment content, see [27].

### Ethical Considerations

As with all psychological treatment, there is a small risk that participants in internet-delivered treatment deteriorate [29]. As an additional safety measure, employees participating in ICBT will respond to questions on suicidal ideation every week and will be monitored weekly for alcohol consumption and mood changes. Among those receiving ICBT, the therapist and employee will meet via weekly video sessions three times during treatment. If a participant deteriorates during ICBT, Alna will offer support face-to-face and via telephone and, if necessary, alternative treatment, and the employer will be informed, consistent with Alna’s standard procedure. During the interviews, the participants will have the opportunity to reflect and reason about their process, which can create awareness of their treatment and perhaps lead to further positive effects of the treatment, which can, in turn, promote healthier behaviors and promote overall well-being.

Another ethical issue is data security. All databases containing personal data will be encrypted to minimize the risk of a breach. In addition, all the servers where data is stored will be encrypted and require a one-time password to be made available for analysis. Furthermore, data will be handled according to the European Union General Data Protection Regulation. All recorded interviews and associated transcripts will be stored on Stockholm University’s encrypted database. The audio files will not contain identifying information (name, workplace, etc). Also, no names or social security numbers will be used in the online survey tool and database (Qualtrics) where self-assessment forms are completed; research participants will identify themselves with a code assigned to them by the researchers.

The issues stated above were addressed in an ethical application to the Swedish Ethical Review authority, which approved (Ref. 2019-04183).

### Planned Analysis

Since the study has a mixed methods design, both quantitative and qualitative methods will be applied.

#### Quantitative Analysis

The quantitative data will be analyzed by using the Reliable Change Index, identifying clinically significant change on an individual level [30]. The significance level to calculate the Reliable Change Index was set at *P*<.05. Analysis of variance or generalized estimating equations will be used to analyze outcome data. Within- and between-group effect sizes also will be calculated.

#### Qualitative Analysis

The interviews will be carried out by the first and second authors. Interview length will vary between 45-60 minutes and will be transcribed ad verbatim. The participants will not be allowed to double-check and confirm the answers given in the interview. The main reason for this is that the participants will receive the questions before the interview and will thus be able to prepare answers. The interviews will be analyzed by using thematic analysis [31] and Grounded Theory [32,33]. Both the first and second authors will code the data. The transcripts from the employees and the employer representatives will be analyzed using inductive thematic analysis. The six steps proposed will be used in the analytic endeavor covering helping and hindering factors [31]. Grounded Theory will be applied to describe the process of attending ICBT in a workplace setting [34]. The coders will have an ongoing discussion about when saturation of the model is met. The focus will be to build a model mirroring the process that the different participants underwent during treatment.

### Attrition Management

Employees who do not complete ICBT will be included in both the quantitative and qualitative analyses to ensure a robust analysis of the outcomes of the treatments. Also, face-to-face non-completers will be included in the analysis. Comparisons between baseline characteristics of the attrition group and treatment completers will be reported. If an employee participating in the qualitative part of the project declines to participate in the interview, an additional participant will be recruited.

## Results

The results from this study will yield an understanding of ICBT in a workplace setting and of whether it could be helpful for employees; an understanding of the employer representative will also be gained.

Recruitment started in April 2020 and is expected to continue until September 2021. Manuscripts for publication will be drafted after that, one focusing on the quantitative outcomes and three focusing on the qualitative analyses. Quantitative results are expected to be submitted at the end of 2021, and the qualitative data will be submitted for publication in spring 2022.

## Discussion

The workplace offers unique opportunities to reach people with problematic alcohol use. Internet-delivered treatment offered within the context of an EAP has several advantages over face-to-face treatment that could stand to benefit both employees (ie, reduced travel time and travel expenses, increased anonymity) and employers (ie, lower production loss). However, proper scientific evaluations are critical before this form of treatment can be meaningfully implemented into such organizations on a broader scale.

### Strengths and Limitations

There are several strengths to the current project. First, although there are a handful of studies conducted on internet-delivered treatment in the workplace [16,17], this is the first study on internet-delivered treatment conducted within the context of an EAP. The current study will, therefore, have important implications for EAPs and similar organizations that are interested in implementing internet-delivered treatment, possibly as an alternative to face-to-face treatment. Further, this is also, to our knowledge, the first study to investigate the internet-delivered treatment of problematic alcohol use using a “mixed methods” approach, ie, applying both quantitative and qualitative methods. Thus, the quantitative evaluations of the treatment will be complemented with qualitative interviews, providing insight into the diversity of experiences and viewpoints among employees and employers involved in the internet-delivered treatment. Finally, outcomes of internet-delivered and face-to-face treatment will be compared in terms of drinking and overall mental health.

Some study limitations are apparent a priori. The quantitative study is a naturalistic one, and the results will indicate possible trends in results, but without the possibility of assessing causality. Also, the qualitative study will consist of a convenience sample from the quantitative cohort, possibly resulting in a biased selection. However, some of the helping and hindering factors associated with delivering ICBT in a workplace context can serve as a starting point for future studies and serve as a foundation for future implementation. Also, the participants might exaggerate treatment gains and positive aspects of the treatment since it is financed by their employer. Special attention will be focused on this aspect during the interviews. Attrition can be a problem in any study investigating treatment effects and is especially common in internet interventions. However, non-completers will be included in the analysis to ensure that attrition is taken into account when reporting outcomes. Another limitation to be taken into account is the probable effect of the assessment procedure. As the Alna assessment with its five separate sessions is quite comprehensive, a positive change in the employee’s consumption, mood, and other symptoms is expected to occur even before the start of treatment. As mentioned, a pre-assessment data collection point has been included to allow evaluation of the impact that the assessment might have.

## Abbreviations

B-PEth: Phosphatidylethanol
CBT: cognitive behavioral therapy
CDT: carbohydrate-deficient transferrin
EAP: employee assistance program
ICBT: internet-based cognitive behavioral therapy
TFLB: timeline followback
